# Recognition of medical error: It is not too late for an open disclosure – a narrative review

**DOI:** 10.1016/j.clinsp.2025.100622

**Published:** 2025-03-18

**Authors:** Ivan Dieb Miziara, Carmen Silvia Molleis Galego Miziara

**Affiliations:** a Full Professor of Legal Medicine, Bioethics, and Occupational Health, Faculdade de Medicina da Universidade de São Paulo, São Paulo, SP, Brazil; b Assistant Professor of Legal Medicine, Faculdade de Medicina ABC, Santo André, SP, Brazil

**Keywords:** Medical error, Open disclosure, Communication skills, Patient safety

## Abstract

A medical error is a preventable adverse effect of medical care. These errors are usually avoidable. They are a vast source of ethical, financial, and even mental problems affecting patients, physicians, and healthcare organizations. The negative impact on patients can be physical, generating sequelae or disability. It could lead to a loss of trust in the patient and his family in the doctor or the hospital where he has been treated.

On the other hand, doctors resist disclosing their errors to the patient, fearing lawsuits and financial losses. Disclosure errors are also an imperative moral duty. In this narrative review, the authors discuss obstacles that exist and prevent an adequate open disclosure, including flaws in medical education, and propose ten steps to proceed with an open disclosure if a medical error occurs.

## Highlights

• Errors during patient care are a huge source of ethical, financial, and even mental problems affecting patients, physicians, and healthcare organizations.
• Open disclosure is a process for ensuring that open, honest, empathic and timely discussions occur between patients and/or their support person(s) and healthcare staff following a patient safety incident.
• Physicians may avoid or hide errors or blame other health professionals.
• Adopting an effective disclosure system, increases the possibility of an agreement between the parties involved, meaning significant savings in financial resources.
• Patients want some attitudes from their doctors: an explicit statement that an error occurred and an apology.

## Introduction

Medical error, generally, could be defined as “a preventable adverse effect of medical care” or “the flawed execution of a sound plan or the wrong plan to achieve a particular aim”.^1^^,^^2^ In Brazil, there is a more specific definition: medical error occurs when an act causes harm to the patient due to negligence, recklessness, or incompetence.

These errors are usually preventable, also called “preventable adverse medical events”, and can occur by commission (what has been done that caused harm to the patient) or omission (what should have been done, but it was not and consequently caused damage to the patient).^3^ Negligent actions differ from other mistakes, but they are a kind of judgment only admissible in a court of law. Negligent actions are often preventable if the physician follows his specialty's guidelines and protocols.

Errors during patient care are a vast source of ethical, financial, and even mental problems affecting patients, physicians, and healthcare organizations. Besides, when an error occurs, it hurts patients and physicians.^4^

The negative impact on patients can be physical, generating sequelae or disability. But it can also be mental due to physical disability (and how to deal with it), some state of mind that generates anger, or a permanent sensation of being wrong. The result of this negative impact on the patient is a loss of trust in doctors and/or hospitals or healthcare institutions. Losing trust causes patients or family members to seek redress (usually financially) from the justice system.

The negative impact on physicians is wicked, too. For example, a physician's depressive symptoms are one of the problems that can arise after an error committed during a patient's treatment.^5^ Despite depressive symptoms being highly prevalent among physicians, it seems that when a medical error occurs, it is aggravated,^5^ or it may manifest for the first time. Worst: a judicial process motivated by the error committed could trigger suicidal thoughts or even actual actions.

Besides, the impact on the mental health of patients and doctors can be acute or chronic. They may appear long after the event has occurred. Minimizing this negative impact requires prompt actions, such as recognizing the error, identifying the type of error, and at least apologizing to a patient and their family in an open disclosure of what has occurred.

Fortunately, regarding mental health problems, some other interventions can reduce their effects,^6^ mainly organizational transformations modification work environment, for example, and also implementing some strategies to prevent errors occurrence.

### What is open disclosure, and why is it necessary?

According to the “Open Disclosure Handbook”, elaborated by the Clinical Excellence Commission of New South Wales in Australia,^7^ “Open disclosure is a process for ensuring that open, honest, empathic and timely discussions occur between patients and/or their support person(s) and health care staff following a patient safety incident”. This commission recommends^7^ that an *Open disclosure is required whenever a patient has been harmed, whether that harm results from an unplanned or unintended event or circumstance or an outcome of an illness or its treatment that has not met the patient's or the clinician's expectation for improvement or cure.*

McLennan et al.^8^ stated that maintaining a humanistic approach “with those who had been harmed” was initiated circa the nineties of the past century at Montreal Hospital. At that time, lawsuits and rising legal costs became more frequent, causing severe damage to physicians and hospitals. In reaction to this situation, the humanistic approach and a caregiving attitude toward the harmed patients significantly reduced complaints and legal costs while improving a better patient-physician relationship.^8^

Improving the relationship between patients and physicians is a key point in this matter. Emphasizing the ethical aspects of medical practice was reinforced in 2006 by the Massachusetts Coalition for the Prevention of Medical Errors' document titled When Things Go Wrong,^9^ which addresses legal, financial, and reputational issues through ethical principles and considerations. This document tries to “re-establish trust by meeting patients' needs and expectations following an incident and improving the quality of care.

Although health practitioners seem to rule by ethical standards and endorse disclosure in principle, “they often do not share information in practice”.^7^ According to some studies, Only 30 % of doctors' harmful errors are disclosed to patients. On the other hand, a physician's reaction to a mistake is a mix of fear and anger.^2^ He experiments on fear and concern about a lawsuit where there may be accusations of malpractice or negligence, seeking compensation for economic losses, and frustration due to perceived injustice. As a result, physicians may avoid or hide errors or blame other health professionals.^2^ The doctor's concern regarding potential legal action is warranted. When a medical error occurs, the possibility of legal action against him and/or the hospital is real. However, fearing legal action against them is not the only motive for not disclosing errors. Some authors argue that causing an error “can assault the practitioner's sense of competence”.^7^ It can trigger defensive psychological responses “which often lead to open communication being avoided altogether or conducted inadequately”.^7^

### Open disclosure, enhanced trust, and financial savings

Adopting an effective disclosure system,^10^ based on the doctor's and hospital's responsibility to the patient care who trusted them for the first time, increases the possibility of an agreement between the parties involved, meaning beyond other outcomes, significant savings in financial resources.

Liang^10^ also says that this quality system “provides for education about the system nature of the error, fulfills the delivery system philosophy of mutual respect, and integrates the patient and his/her family as partners in the error reduction enterprise”.

In this way, detection methods to prevent and reduce errors in a medical setting advance in the 21st century. Khan et al.^11^ stated that “medical record review-based surveillance methods” are ten times more efficient than simple incidence-reporting systems.

Besides the physician's attitude, there are other problems in dealing with an error. The 1999 release of the American Institute of Medicine's document To Err is Human brought the need to deal with it to the forefront.^12^

In the meantime, in Brazil, the processes of judicialization of medicine due to medical errors are becoming more frequent.^13^ Most incidents arise from inappropriate medical conduct, which can harm the patient. However, some occurrences are unjustifiable and contribute to a strained medical-patient relationship.

Justifiable or not, as we said, an accusation of medical error can produce deleterious effects on doctors and patients. If the doctor or the hospital pleads guilty, the cost of the process could be unbearable. If the doctor or the hospital pleads not guilty, the patient or his family claims he was wronged and “the corporative system won one more time”.

However, as medicine evolved, twenty-five years after the publication of To Err is Human,^9^ which represented a turning point in patient-safety strategies, many highly effective interventions have since been developed, most of them regarding hospital-acquired infections and medication safety, with a variable impact on the expected outcome of these measures.^14^ Otherwise, errors and/or adverse events continue, such as not telling the patient the truth in situations like that. It makes us wonder if the root of the problem might not be in the doctor's training during (and after) medical graduation.

Thus, this narrative review aims to analyze better strategies to deal with and disclose medical errors, if they occur, and proposes some of them to minimize these deleterious effects regarding patient safety, save costs to doctors and hospitals, and prevent case courts.

## Methods

The authors made a search query analyzing results from medical journal articles with keywords such as “recognition of medical errors”, “patient safety”, “medical liability”, and “medical litigation” in databases such as Ovid, Embase, MEDLINE, Pubmed, Cochrane Library, Scopus, Scielo, and Google Scholar from 2000 to 2025. The authors chose 28 articles for this narrative review, all of which we considered essential to explain the author's point of view.

## Discussion

When an error occurs, there are diverse ways to distinguish the types of incidents and the terminology used. Examples include medical errors, patient safety incidents, and adverse events.^15^ In this context, regardless of the causes that lead to the mistake, recognizing failure is integral to repairing the damage caused. First, it is a moral duty, and as said by Hammami et al.,^16^ when an error occurs, two actions should be considered: reporting it to the healthcare system (and through it to potential future patients) and disclosing it to the patient involved. These actions can prevent a lawsuit against the doctor or hospital from being considered or could result in a mutually satisfactory agreement. It is crucial because not facing a lawsuit means saving resources for the doctor or hospital organization. However, promptly identifying an error is one of the most critical steps in this process. Several countries have guidelines for healthcare providers to disclose medical errors to patients.^17^

### Saving money and ethics

Financial savings are an essential aspect when it comes to recognizing a mistake made by a doctor, but it is not the main one. As mentioned above, disclosing an error is an ethical imperative sustained by various moral theories. The primary principle is that the patient should be informed wholly and promptly whenever an error occurs. The principles behind the professional duty of truth-telling include respecting patient autonomy and trust, as stated by Matlow et al., and as the authors said before.^18^^,^^19^ The speed at which an error is recognized and reported affects the correction of the error and the safety and well-being of the patient.

### Strategy to promote excellence

Historically, since Harvey Cushing used his strategy to promote excellence in the medical profession and encourage the education of publishing successes and failures, it became a normative way of good practice.^9^ There were opposing views. In the past century, “silence strategies” prevailed. These strategies, fortunately, “decades later have this guarded approach been called into question”.^9^

There are two distinct aspects the authors can identify in the recognition of an error and the investigation of its causes. The first is when the error affects only one patient. The other is when an error affects multiple patients. When a single patient is concerned, the blame falls on the physician responsible for the care, as he is closest to the patient and his family members. This error or adverse event can occur due to recklessness, negligence, or malpractice. Errors occur or persist because they are not immediately apparent. Concerns may initially be raised by a clinician, technicians, or even the patient themselves, as noted by Chafe et al.^17^

For example, when a laboratory or diagnostic testing error has occurred (e.g., wrong altered blood count requiring transfusion or glycemia with incorrect values, meaning unnecessary insulin doses), the first step is determining whether the resulting diagnostic report affected the clinician's decision-making. It could be an isolated event or involve multiple patients (e.g., patients with wrong altered blood count requiring blood transfusion). The first steps in these situations are crucial. An expert clinician or health care team should assess the appropriateness of the patient care provided, potential outcomes, and decisions made by the health staff.^14^ The authors recommend this strategy even if the error affects just one patient.

### Strategies proposed for open disclosure and patient safety

Painter et al.^20^ investigated if written or verbal disclosure of serious events led to increased malpractice claims or higher compensation costs. The authors concluded that these procedures have no interference in the outcome, e.g., “was not associated with an overall increase in malpractice claims”. Although the authors had analyzed 15,028 serious events disclosures and 1302 total malpractice claims on a universe of 1587,842 patients, the authors propose that written or verbal disclosure of serious events is critical. At least this procedure signals to the patient (or the judge) that the doctor acted in good faith and showed honesty and concern with his patient.

In any situation, showing errors is a complex process. So, it is necessary for one involved in this process regarding patient safety to develop a comprehensive approach. When facing only one individual, the patient's perspective is clear. Patients want some attitudes from their doctors:^9^

1) An explicit statement that an error occurred.
2) To be told what the error was.
3) To be told why the error occurred.
4) To know what will be done to prevent recurrences.
5) An apology.

It is important to note that “the literature and the lay press may lead some to think clinicians are making more errors than ever before”.^20^ Besides, although errors in medicine are not a new phenomenon, the expectation that a clinician reports his error to the patient harmed is new;^12^ it is a new moral duty. As stated by Hall^21^ and as the authors stated previously,^22^ telling the truth about an error is faithful to the patient. It is a component of the more comprehensive value of fidelity, of being true to one's patient. Some authors have suggested that clinicians should prioritize informing patients about errors when they occur, as it can lead to positive outcomes.

According to the “Open Disclosure Handbook, elaborated by the Clinical Excellence Commission de New South Wales in Australia”,^7^ there are five essential elements of open disclosure: an apology, a factual explanation of what happened, an opportunity for the patient to relate their experience, a discussion of the potential consequences, and an explanation of the steps being taken to manage the event and prevent a recurrence. In addition to these essential elements, adequate open disclosure also includes:^7^ *Acknowledging the patient and/or their support person(*s*) when things go wrong; listening and responding appropriately when the patient and/or their support person(*s*) relate their experiences, concerns, and feelings; the opportunity for the patient and/or their support person(*s*) to ask questions and to have those questions answered; providing support for patients and their support person(*s*) and health care staff to cope with the physical and psychological consequences of what happened.*

### Disclosure and apology laws

Many authors propose the creation of specific laws, known as “disclosure and apology laws”.^23^ Guillod quotes the recommendation of the Council of Europe on managing patient safety and preventing adverse events in health care, which “stressed the fact that legislation constitutes one of the most important regulatory mechanisms in healthcare. In other words, laws can contribute to a change in professional cultures”.^23^ However, the author emphasizes that “the traditional medical culture” and the legal system are “short-sighted”. He states doctors do not disclose errors because they are “afraid of being held liable”.^23^ They also misunderstand apology as “an admission of guilt or liability”, whether we know nowadays that the injured patient wants more explanation about how the unfortunate event occurred and some apology rather than a simple “financial compensation”.^10^^,^^21^ In this aspect, as a new moral duty, education about open disclosure is essential to implement the better strategies proposed.

### Education and training communication skills

Guillod^23^ suggests training inexperienced doctors to enhance communication and awareness of clinical risk management, along with implementing Critical Incident Reporting Systems in healthcare settings. Hospitals and all the health staff should adopt tools such as M&M reviews, quick alerts, dashboards, safety checklists, guidelines, recommendations, etc., to improve patient safety. Besides, systematically disclosing errors to injured patients and/or their families is fundamental. It increases transparency and builds trust.

Corroborating this point of view, Swinfen et al.^24^ reviewed the literature on this topic. The authors produced a descriptive and cross-sectional study with 132 fifth-year medical students. They concluded a “dire need for more frequent experiential training in disclosing medical errors in undergraduate medical education”.

Kaldjian et al.^25^ state that “disclosing errors are poorly understood” despite being an essential part of patient care. They conducted a multicenter study with 538 participants from three medical schools in the United States. The objective of their multicentric study (joining the faculties of medicine of Iowa University, Yale University, and Penn State University) was to survey physicians and trainees about their practices and attitudes regarding error disclosure to patients. They refined their survey using some measures according to the type of event and attitudes in front of these events, e.g., “actual error disclosure; hypothetical error disclosure; attitudes toward disclosure”. Their conclusions were like the Swinfen et al.^24^ conclusions: that there exists “a gap between physician's attitudes and practices regarding error disclosure” and that “willingness to disclose errors was associated with higher training level and a variety of patient-centered attitudes”. However, the most worrying thing is that this willingness to disclose errors “was not lessened by previous exposure to malpractice litigation”.^25^ In other words, not even having suffered a lawsuit could change the doctor's attitude and behavior toward the patient when an error occurs. Although doctors worldwide tend to avoid error disclosure^21^ because of some fears of liability, or, as stated by Detsky et al.,^26^ “ethics say yes, instinct says no”, we should expect that having suffered a previous lawsuit served as motivation for the doctor to improve his ethical principles when dealing with patients, but unfortunately, it did not occur.

Sukalich et al.^27^ conducted a study in 2014 that aimed to “determine whether a standardized patient encounter and self-guided tutorial would improve first-year residents' self-efficacy for disclosing medical errors”. The authors concluded that a “timely, explicit, and empathetic” disclosure to patients and their families of failure to receive treatment or physical harm due to medical errors is significant to maintaining trust in patient-centered medical care. So, these standardized encounters and self-guided tutorials must be implemented as a good strategy for better practices.

### Is a change in the legal system possible?

Otherwise, Guillod^23^ also suggests other measures, such as abandoning “the traditional regime of civil liability based on individual faults and setting up a no-fault compensation scheme” and “banning ex officio criminal proceedings against health professionals when patients have been hurt in the course of medical treatment”. A no-fault compensation is possible in terms of the agreement, but the latter is impractical in Brazilian society. Despite the correction of the author's arguments, in short, that “the law isolated is less effective in preventing medical error”, it is impractical in our country because, in our legal system, it is not possible (with some exceptions) for the opening of an “ex-officio” process. This type of measure demands an alteration in our Penal Code through the legislative system.

The Brazilian Penal Code,^28^ for example, in its articles 129 and 121, predicts that if a doctor causes harm to a patient by negligence, recklessness, or malpractice, or by a non-observance of professional technical rules, he deserves more serious punishment. Otherwise, in Brazil, in civil law, malpractice complaints often reach the court by a demand from the patient's lawyer for free (if the demandant cannot afford a lawsuit). Free Justice is a Brazilian institution that benefits many people who could not access the justice system without it. As a side effect of this constitutional right, many take advantage of this facility to initiate lawsuits against doctors, increasing (sometimes unfairly) the judicialization of Brazilian medicine.

### What the doctor has to do if an error occurs (ten measures to take into account)

In short, based on the discussion above and in the search for the literature that is disposable, we propose some measures:

1) Act quickly and honestly;
2) Reunite all the health staff and identify what type of error occurred; identify flaws in the patient safety program;
3) If a patient safety program does not exist in the institution, create one;
4) Explain to the patient and his surrogate what happened, ask their questions about the incident honestly;
5) Do not blame other professionals: you are directly responsible for patient safety;
6) Explain that the patient will receive all the health support he needs to minimize the damage caused;
7) Apologizing for the damage caused is essential;
8) Tell them what measures to prevent another mistake from being taken;
9) Initiate an education program in open disclosure to residents and trainees;
10) If necessary, call the hospital's lawyers or the legal department to make an agreement.

### Final considerations

In summary, to counter this state of things and avoid judicialization, the doctor's honesty, recognition of the error (if applicable), open disclosure, apologies, and a good relationship with his patient and their family can be an essential path. The sooner these measures are adopted, the better. But it is never too late to learn, be ethical, and save costs.

## Conflicts of interest

The authors declare no conflicts of interest.
